# Loss-of-Function Mutation of Soybean R2R3 MYB Transcription Factor Dilutes Tawny Pubescence Color

**DOI:** 10.3389/fpls.2019.01809

**Published:** 2020-01-31

**Authors:** Fan Yan, Stephen M. Githiri, Yajing Liu, Yu Sang, Qingyu Wang, Ryoji Takahashi

**Affiliations:** ^1^ College of Plant Science, Jilin University, Changchun, China; ^2^ Horticulture Department, Jomo Kenyatta University of Agriculture and Technology, Nairobi, Kenya; ^3^ NARO Institute of Crop Science, Tsukuba, Japan

**Keywords:** soybean, MYB transcription factor, flavone synthase II, pubescence color, *Td* gene

## Abstract

Pubescence color of soybean is controlled by two genes, *T* and *Td*. In the presence of a dominant *T* allele, dominant and recessive alleles of the *Td* locus generate tawny and light tawny (or near-gray) pubescence, respectively. Flavones, responsible for pubescence color, are synthesized *via* two copies of flavone synthase II genes (*FNS II-1* and *FNS II-2*). This study was conducted to map and clone the *Td* gene. Genetic and linkage analysis using an F_2_ population and F_3_ families derived from a cross between a Clark near-isogenic line with light tawny pubescence (genotype: *TT tdtd*) and a Harosoy near-isogenic line with tawny pubescence (*TT TdTd*) revealed a single gene for pubescence color around the end of chromosome 3. Genome sequence alignment of plant introductions revealed an association between premature stop codons in Glyma.03G258700 (R2R3 MYB transcription factor) and recessive *td* allele. Cultivars and lines having near-gray or light tawny pubescence and a gray pubescence cultivar with *td* allele had premature stop codons in the gene. These results suggest that Glyma.03G258700 corresponds to the *Td* gene. It was predominantly expressed in pubescence. Compared to a tawny pubescence line, a near-isogenic line with *td* allele produced extremely small amounts of transcripts of Glyma.03G258700, *FNS II-1,* and *FNS II-2* in pubescence. The promoter of *FNS II-1* and *FNS II-2* shared *cis*-acting regulatory elements for binding of MYB proteins. These results suggest that the wild type of Glyma.03G258700 protein may bind to the promoter of *FNS II* genes and upregulate their expression, resulting in increased flavone content and deeper pubescence color. In contrast, mutated Glyma.03G258700 protein may fail to upregulate the expression of *FNS II* genes, resulting in decreased flavone content and dilute pubescence color.

## Introduction

Pubescence color of soybean [*Glycine max* (L.) Merr.] is controlled by two genes (*T* and *Td*) (Palmer et al., 2004). The *T* gene has a major effect on pubescence color; dominant *T* and recessive *t* allele produce tawny and gray pubescence, respectively. Further, the *T* gene controls coloration of the seed coat and hypocotyl (Palmer and Payne, 1979; Palmer et al., 2004; Murai et al., 2016). The *T* gene encodes a flavonoid 3ʹ-hydroxylase and hydroxylates flavonoids at the 3ʹ-position of the B-ring to generate dihydroxylated flavonoids (Buttery and Buzzell, 1973; Toda et al., 2002). The entire coding region of *F3'H* was cloned from a pair of near-isogenic lines (NILs) for the *T* gene, To7B (*TT*), and To7G (*tt*). They differed by one base deletion in To7G, resulting in a short polypeptide lacking the consensus sequence GGEK and heme-binding domain (Toda et al., 2002).

Bernard (1975) reported another gene, *Td,* affecting pubescence color. In the presence of the dominant *T* allele, dominant and recessive alleles of the *Td* locus generate tawny and light tawny (or near-gray) pubescence, respectively. Recessive *t* allele generates gray pubescence color irrespective of the allele of the *Td* locus (*tt TdTd* or *tt tdtd*) (Bernard, 1975). In contrast to the *T* gene, *Td* gene affects only pubescence color (Palmer and Payne, 1979; Palmer et al., 2004).

Existence of different flavonoids is responsible for variation of tissue coloration in soybean. Iwashina et al. (2006) characterized flavonoids in soybean pubescence. A large amount of luteolin aglycone (flavone with 3ʹ4ʹ-dihydroxylation) was extracted together with small amounts of apigenin aglycone (flavone with 4ʹ-hydroxylation) and luteolin derivatives from tawny pubescence. Luteolin aglycone comprised the largest portion (95–96%). From gray pubescence, a large amount of apigenin aglycone was extracted together with small amounts of luteolin aglycone, apigenin 7-*O*-glucoside, and apigenin derivatives. Apigenin aglycone was most abundant (90%). Thus, the hydroxylation pattern of the B-ring is associated with the dominance of the *T* gene. From light tawny pubescence of Clark-*td*, a Clark NIL with *td* allele, three compounds identical to those in tawny pubescence were extracted. However, compared to tawny pubescence, the amount of luteolin aglycone was reduced to half. In addition, two high performance liquid chromatography (HPLC) peaks corresponding to isoflavonoids were occasionally found in light tawny pubescence (Iwashina et al., 2006). These results suggest that the *Td* gene might be involved in the biosynthesis of flavones. Flavone glycosides exist in vacuoles in pubescence whereas flavone aglycones exist outside the cell surface, and they belong to so-called surface flavonoids (Iwashina et al., 2006). Biological roles of surface flavonoids remain to be investigated (Wollenweber, 1994). Most pigments in pubescence could not be extracted from pubescence, suggesting that flavone derivatives are highly polymerized (Iwashina et al., 2006). In soybean, flavones predominantly exist in pubescence and are quite rare in other tissues (Iwashina et al., 2006).

Plants have evolved two independent enzyme systems to synthesize flavone using the same substrates, flavone synthase (FNS) I and II (Martens and Mithofer, 2006). Both enzymes never occur side by side in the same organism. FNS is a soluble 2-oxoglutarate- and Fe^2+^-dependent dioxygenase, occurs in Apiaceae, whereas FNS II, a membrane-bound cytochrome P450 monooxygenase, is more widespread among plant species. Soybean has two functional copies of FNS II gene, *FNSII-1* (Glyma.12G067000) and *FNSII-2* (Glyma.12G067100) with 93% amino acid identity, with an approximately 8 kb distance on chromosome 12 (Fliegmann et al., 2010; Jiang et al., 2010).

Genome-wide association studies (GWASs) using a total of 1,402 soybean genotypes revealed a significant signal associated with pubescence color at 47,244,893 bp on chromosome 3, presumably corresponding to the *Td-td* locus (Wen et al., 2015). In addition, GWAS of a total of 12,360 accessions suggest a signal associated with the *Td-td* locus around the end of chromosome 3 (Bandillo et al., 2017). However, the identity and nature of the *Td* gene has not yet been clarified. This study was conducted to map and clone the *Td* gene responsible for flavone content and deepening/diluting of pubescence color in soybean.

## Materials and Methods

### Plant Materials

Plant materials used in this study are listed in **Table 1**. Pedigree and pubescence color information was obtained from the USDA GRIN database (https://www.ars-grin.gov/npgs/). Soybean NIL of US cultivar Clark, L66-260 with light tawny pubescence (Clark-*td*; genotype *TT tdtd*) was crossed with L66-707, NIL of Canadian cultivar Harosoy with tawny pubescence (Harosoy-*T*; *TT TdTd*) in 2004. Flowers of Clark-*td* were emasculated 1 day before opening and pollinated with pollen of Harosoy-*T*. Hybridity of F_1_ plants was ascertained by the existence of tawny pubescence color. The NILs were developed by backcrossing the pubescence color traits five times into Clark or Harosoy backgrounds (Bernard et al., 1991). In addition, five US cultivars with light tawny or near-gray pubescence (Grant, Korean, Cloud, Kingwa, and Sooty) as well as a gray-pubescence US cultivar Seneca, based on pedigree information, presumably having the recessive *td* allele (Bernard, 1975) were used for genotyping. Seeds of the NILs and the cultivars were provided by the USDA Soybean Germplasm Collection.

**Table 1:** Plant materials used in this study.

Cultivar or line name	Line designation	Origin	Genotype	Pubescence color	Purpose
Harosoy-*T*	L66-707	Harosoy (6) x Clark	*TT TdTd*	Tawny	Genetic analysis and cDNA cloning
Clark-*td*	L66-260	Clark (6) x PI 91160 collected from China	*TT tdtd*	Light tawny	Genetic analysis and cDNA cloning
Grant	–	Lincoln x Seneca	*TT tdtd*	Light tawny	Genotyping
Korean	–	Collected from North Korea	*TT tdtd*	Light tawny	Genotyping
Cloud	–	Selected from PI 16790 collected from China	*TT tdtd*	Near-gray	cDNA cloning
Kingwa	–	Selected from Peking	*TT tdtd*	Near-gray	Genotyping
Sooty	–	Selected from Cloud	*TT tdtd*	Near-gray	Genotyping
Seneca	–	Selected from FC 03654 collected from China	*tt tdtd*	Gray	Genotyping
PI 157421	–	Collected from South Korea	*TT tdtd*	Near-gray	Next-generation sequencer (NGS) data analysis
PI 84631	–	Collected from South Korea	*TT tdtd*	Near-gray	NGS data analysis
PI 549046	–	Collected from China	*TT tdtd*	Near-gray	NGS data analysis
Bay	–	Selected from York x R62-550 (Essex x *G. soja*)	*tt TdTd*	Gray	NGS data analysis
Williams 82	–	Williams (7) x Kingwa	*TT TdTd*	Tawny	cDNA and DNA cloning

### Genetic Analysis

A total of 120 F_2_ seeds together with 30 seeds each from the parents were planted in a field at the NARO Institute of Crop Science (36°06′ N, 140°05′ E) on 14 June 2005. N, P, and K were applied at 3.0, 4.4, and 8.3 g m^−2^, respectively. Thirty seeds each from 98 F_3_ families and parents were planted in the same field on 8 June 2007. Spaces between rows and plants were 70 and 10 cm, respectively. Genotype of F_2_ plants was determined by pubescence color segregation of the F_3_ families.

### Linkage Mapping

A total of 94 F_2_ plants were randomly selected and used for linkage mapping because the PCR plates and electrophoresis apparatus are designed for multiples of 96 samples (2 parents and 94 F_2_ plants). Total DNA was extracted from trifoliate leaves of the parents and each of the F_2_ plants by the CTAB method (Murray and Thompson, 1980). Pubescence color was scored in each of the F_2_ plants and F_3_ families. Based on pubescence color in F_2_ and F_3_ generations, 24 F_2_ plants fixed for tawny pubescence and 24 F_2_ plants fixed for light tawny pubescence were identified. Two bulked DNA samples (one for tawny pubescence and another for light tawny pubescence) were obtained by mixing 10 μl from each of the selected plants and were subjected to bulked segregant simple sequence repeat (SSR) analysis. Polymorphic SSR markers in Harosoy x Clark (Nebraska) population (Cregan et al., 1999) were used for analyzing variation between the two bulked DNA samples. PCR conditions were similar to those in a previous report (Rojas Rodas et al., 2014). Variation between the two bulked samples was used to determine which chromosome harbored the pubescence color gene. Base on the results, SSR markers on chromosome 3 (Song et al., 2004) were then tested for variation among the 94 F_2_ plants. The linkage map was constructed using MAPMAKER/EXP version 3.0 (Lander et al., 1987) with the Kosambi function and the threshold logarithm of odds (LOD) score of 3.0. To fill the gap in the linkage group, SSRs were screened and an SSR marker designated as Gm03:45226162-45226406 was constructed using the Simple Sequence Repeat Identification Tool (http://archive.gramene.org/db/markers/ssrtool).

### Next-Generation Sequencer Data Analysis

Based on information provided by Zhou et al. (2015), whole genome sequences of three USDA plant introductions with near-gray pubescence (PI 157421: SRR1533292; PI 84631: SRR1533280; PI 549046: SRR1533204) were downloaded using the DRA Search of the DNA Data Bank of Japan (http://ddbj.nig.ac.jp/DRASearch/query?keyword=PI+196166&show=20) (**Table 1**). The genome sequences together with our next-generation sequencer (NGS) data of US cultivar Bay with gray pubescence were uploaded to the Integrative Genomics Viewer (http://software.broadinstitute.org/software/igv/) (Robinson et al., 2011) and were compared with those of Williams 82 having tawny pubescence.

### Molecular Cloning

Total RNA was isolated from trifoliate leaves (100 mg) of Harosoy-*T*, Clark-*td,* Cloud, and Williams 82 using the Spin Column Plant Total RNA Purification Kit (Sangon Biotech, Shanghai, China) according to the manufacturer's instructions. cDNA was synthesized by reverse transcription polymerase chain reaction of 5 μg of total RNA using the PrimeScript II 1st Strand cDNA Synthesis Kit (Takara, Dalian, China) and an oligo (dT) primer following the manufacturer's instructions. cDNA was amplified by end-to-end PCR using primers (**Table 2**) that were based on the genome sequence of US cultivar Williams 82 deposited in the soybean genome database (Phytozome, https://phytozome.jgi.doe.gov/pz/portal.html#). The total volume of 50 μl PCR mixture contained 10 pmol of each primer, 1 × PrimeSTAR Max Premix (Takara), and 200 ng cDNA. The PCR program was 35 cycles of 10 s denaturation at 98°C, 15 s annealing at 56°C and 1 min extension at 72°C. PCR was performed in an Applied Biosystems 9700 thermal cycler (Thermo Fisher Scientific, Waltham, USA). Each target band was TA cloned into the plasmid vector pMD18-T (Takara). The plasmids were sent to the Sangon Biotech for sequencing.

**Table 2:** PCR primers used in this study.

Purpose	Target gene	Forward primer (5'–3')	Reverse primer (5'–3')
Simple sequence repeat (SSR) analysis (Gm03:45226162-45226406)	–	GGAGGATTAAATTCGCTCCA	TTCGGTGAGGTCTCTAAAAGAT
cDNA cloning	Glyma.03G258700	TAAGGTGCTCGATCTGCTT	AATATGTTCACGTGCTAGCA
DNA cloning	Glyma.03G258700	TAAGGTGCTCGATCTGCTT	ATGGGTATTCCAGTAGTTCT
		ACTGAGATGGATGAACTATC	TGCTAGCACGTGAACATATT
	2nd exon of Glyma.03G258700	AATCAGGGTTAAGGCTTTAT	AATATGTTCACGTGCTAGCA
Derived cleaved amplified polymorphic sequences (dCAPS) analysis for single-nucleotide polymorphism (SNP)	Glyma.03G258700	AAAGGGTCAGAGAAGACCTTA	TCCAATATTGTCACTTACCGC
dCAPS analysis for indel	Glyma.03G258700	AGACAATGGTGGGATTGGGA	AATATGTTCACGTGCTAGCA
Real-time PCR	Glyma.03G258700	GGAAAAAGTTGCCGACTGAG	TGGATCCGTTCCTTGATTTC
	*GmFNSII-1*	AGGTCCAGTTAACTCGGAACCAT	GCTTCACACACTAATTGCGTGT
	*GmFNSII-2*	AGGTCCAGTTAACTCGGAACCAT	GCTTCACACACTAATCGCTTGT
	*actin 1*	GTCCTTTCAGGAGGTACAACC	CCACATCTGCTGGAAGGTGC

Genomic DNA was extracted from trifoliate leaves of Grant, Korean, Cloud, Kingwa, Sooty, Harosoy-*T*, Clark-*td*, and Williams 82 by the CTAB method. Entire genomic fragment was amplified from Williams 82 whereas the second exon was amplified from the other cultivars using the PCR primers in **Table 2**. PCR mixture contained 10 pmol of each primer, 1 × PrimeSTAR Max Premix, and 50 ng genomic DNA in a total volume of 50 μl. The PCR program was 35 cycles of 10 s denaturation at 98°C, 5 s annealing at 56°C, and 10 s extension at 72°C. The PCR products were sent to Sangon Biotech for direct sequencing.

### Sequencing Analysis

Nucleotide sequences of both strands were determined with the BigDye terminator cycle method using an ABI 3730XL (Thermo Fisher Scientific). Nucleotide sequences and the putative amino acid translations were analyzed with the BLAST program (Altschul et al., 1997). Nucleotide and amino acid sequences were aligned using ClustalW (http://clustalw.ddbj.nig.ac.jp/top-j.html) with default settings. The amino acid alignment was used to construct a phylogenetic tree of MYB genes related to flavonoid biosynthesis (**Additional File 1: Table S1**) using the neighbor-joining method with MEGA version 10.0.5 (http://www.megasoftware.net/) (Kumar et al., 2018). Bootstrap test of 1,000 replications was performed. Nucleotide sequences of the putative promoter region (up to 1,500 bp upstream from the start codon) of two *FNSII* genes, *GmFNSII-1* (Glyma.12G067000) and *GmFNSII-2* (Glyma.12G067100) of Williams 82 were downloaded from Phytozome. *Cis*-acting regulatory elements of these genes were investigated using New PLACE, a database of plant *cis*-acting regulatory DNA elements (https://sogo.dna.affrc.go.jp/cgi-bin/sogo.cgi?lang=en&pj=640&action=page&page=newplace), using default settings (Higo et al., 1999).

### Derived Cleaved Amplified Polymorphic Sequences Analysis

Two pairs of PCR primers containing mismatched nucleotides were designed to detect a single-nucleotide polymorphism (SNP) existing in Clark-*td* and one base deletion in Cloud. The SNP of Clark-*td* was expected to result in the presence of the restriction site of *Bsr*BI in the amplicons. The deletion in Cloud was expected to result in the presence of the restriction site of *Eco*RV in the PCR products. The total volume of 25 μl PCR mixture contained 10 pmol of each primer, 1 × PrimeSTAR Max Premix, and 50 ng genomic DNA. The PCR program was 35 cycles of 10 s denaturation at 98°C, 5 s annealing at 56°C, and 10 s extension at 72°C. The PCR products were digested with *Bsr*BI or *Eco*RV, and the digests were separated on 8% nondenaturing polyacrylamide gels. After electrophoresis, the gels were stained with ethidium bromide and the DNA fragments were visualized under UV light.

### Quantitative Real-Time PCR

Tissue samples were collected in three replicates. Pubescence was sampled with razor blades from pods and stems of Harosoy-*T* and Clark-*td* at the R3 stage (Fehr et al., 1971). Petals were collected on the day of opening from Harosoy-*T*. Leaves, stems, roots, root nodules, and immature seeds were sampled from Harosoy-*T* at the R3 stage. Total RNA was extracted from 50 mg of pubescence, and 100 mg of the other tissue samples. cDNA was synthesized by reverse transcription polymerase chain reaction of 1 μg of total RNA using the PrimeScript RT reagent Kit with gDNA Eraser (Perfect Real Time) (Takara) and an oligo (dT) primer following the manufacturer's instructions. Six concentration gradients (0, 1/2, 1/4, 1/8, 1/16, 1/32) were used to set up a standard curve. The testing samples of cDNA were diluted five times. Three biological replications and three technical repetitions were tested for quantification. The real-time PCR mixture contained 1 × TB Green *Premix Ex Taq* (Tli RNaseH Plus) (Takara), 10 pmol of each primer, 1 × ROX reference dye, 50 ng of cDNA, and water to a final volume of 20 μl. The PCR was performed using the StepOnePlus Real-Time PCR instrument (Thermo Fisher Scientific). The initial 30 s denaturation at 95°C was followed by 40 cycles of 5 s denaturation at 95°C and 30 s annealing at 60°C. The expression level of the target gene was normalized using soybean *actin 1* gene (GenBank accession number: J01298). The PCR primers are listed in **Table 2**.

## Results

### Inheritance of Pubescence Color

From a total of 120 F_2_ seeds planted, 104 plants grew normally. Some of the F_2_ plants were difficult to classify into tawny or light tawny pubescence class as previously reported (Bernard, 1975) so F_3_ progeny tests were performed. A total of 98 F_2_ plants were randomly selected, and their F_3_ seeds were planted in the field. Ninety-eight F_3_ families were segregated into 24 families fixed for tawny pubescence, 50 segregating families, and 24 families fixed for light tawny pubescence. The segregation fitted to a single gene control model of 1:2:1 (χ^2^ = 0.04, *P* = 0.98), in accordance with the previous report (Bernard, 1975).

### Mapping of Pubescence Color Gene

**Figure 1:**
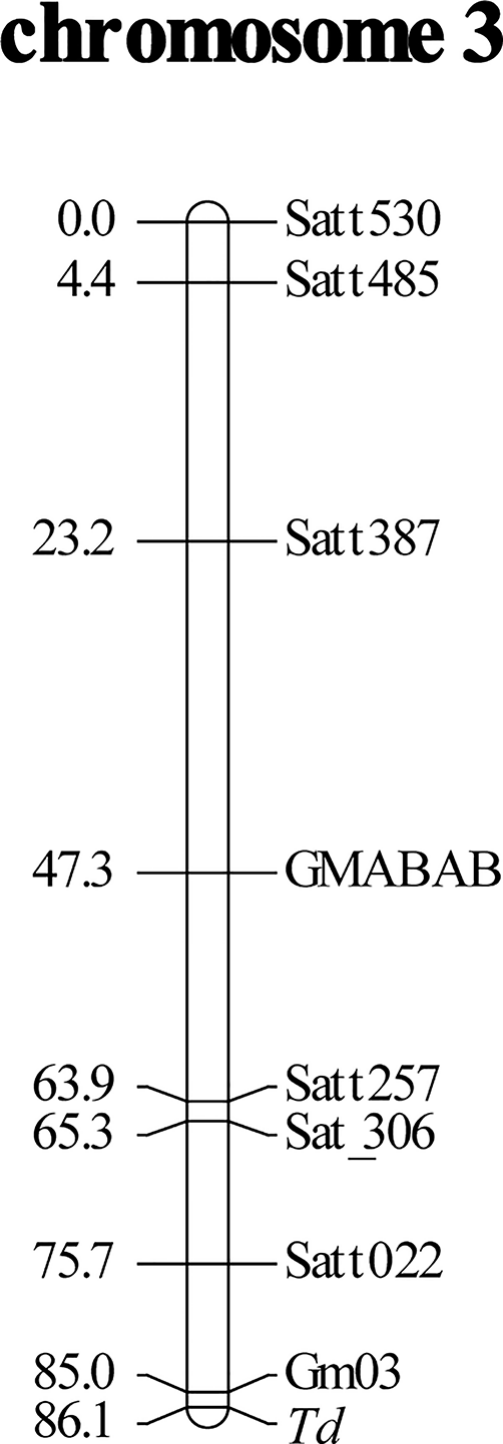
Linkage mapping of soybean pubescence color gene *Td* using an F_2_ population derived from a cross of near-isogenic lines, Clark-*td* x Harosoy-*T*. Distances of markers (cM) from the top of linkage group are shown on the left. Gm03 is an abbreviation of a newly developed simple sequence repeat (SSR) marker, Gm03:45226162-45226406.

Bulked segregant analysis suggested that the *Td* gene was located in chromosome 3. Seven SSR markers in chromosome 3 had good separation and were used for linkage mapping. The *Td* gene was mapped toward the end of chromosome 3. To fill the gap between the *Td* gene and Satt022, a new SSR marker Gm03:45226162-45226406 was designed (**Table 2**). The primers were constructed flanking nine repeats of the AT motif. The *Td* gene was mapped 1.1 cM away from the marker toward the chromosome end, generating a linkage group spanning 86.1 cM (**Figure 1**).

### Candidate Gene Identification

**Figure 2:**
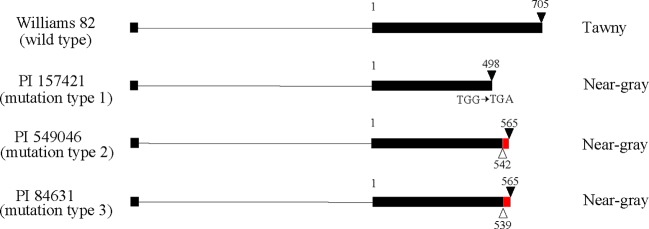
Gene structure of Glyma.03G258700 in Williams 82 with tawny pubescence and three plant introductions with near-gray pubescence. Exons and introns are shown by boxes and lines, respectively. White and black triangles indicate deletions and stop codons, respectively. Red box represents a region where frameshift mutation occurred due to deletion. Numbers indicate nucleotide position starting from second exon.

There are 71 genes (Glyma.03G257900 to Glyma.03G254900) from Gm03:45226162-45226406 to the chromosome end in Williams 82. Based on genome sequence comparison with Williams 82 (*Glycine max* Wm82.a2.v1), plant introductions with near-gray pubescence (PI 157421, PI 84631, and PI 549046) had mutations in the coding region of Glyma.03G258700, which is annotated as a MYB transcription factor (**Figure 2**). In PI 157421, base substitution from TGG to TGA generated a stop codon in the coding region. In PI 549046 and PI 84631, a single-base deletion occurred at different positions 3 bp apart from each other in the second exon, resulting in premature translation termination. These results suggest that Glyma.03G258700 might correspond to the *Td* gene.

However, according to the reference genome of Williams 82, cDNA of Glyma.03G258700 is truncated and lacks a start codon (**Figure 2**). Genome sequence alignment of the plant introductions and cultivar Bay indicated that no NGS reads were allocated to a 58-nucleotide region (Gm03:45302852 to Gm03:45302909) corresponding to the middle of the first exon in all genotypes (**Additional File 1: Figure S1**). We designed a forward primer in the upstream region to amplify the entire coding region of Glyma.03G258700 (**Table 2**, **Additional File 1: Figure S1**, and **Additional File 3: Figure S2**).

### Gene Cloning

**Figure 3:**
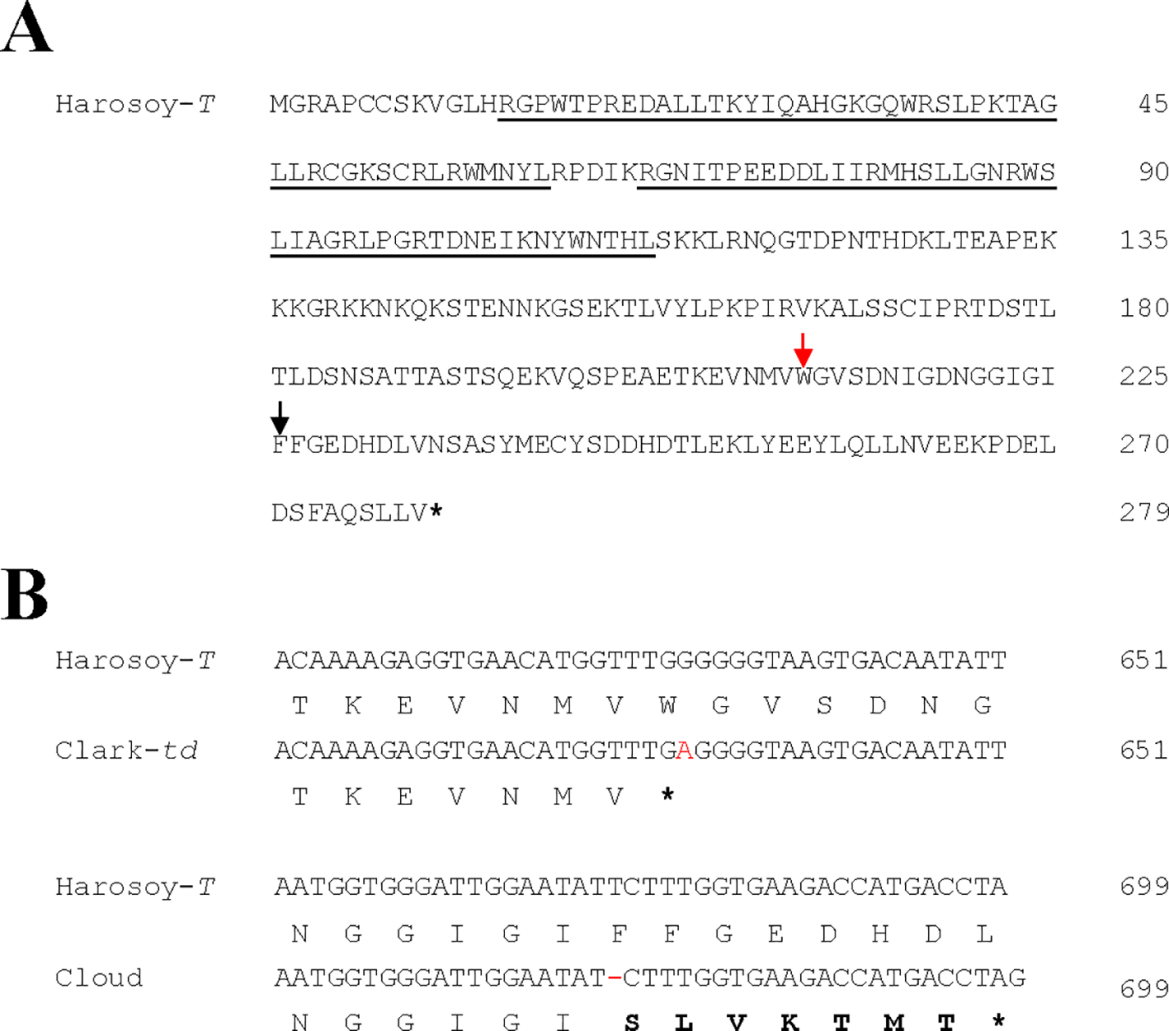
Nucleotide and amino acid polymorphisms of the soybean gene Glyma.03G258700 in Harosoy-*T*, Clark-*td*, and Cloud. Asterisk represents stop codon. **(A)** Amino acid sequence of Harosoy-*T*. The position of single-nucleotide polymorphism (SNP) and indel found in Clark-*td* and Cloud are indicated by red and black arrows, respectively. MYB DNA-binding domains are underlined. **(B)** Alignment of partial cDNA and amino acid sequences. The SNP in Clark-*td* and an indel in Cloud are shown in red font. The altered amino acids caused by frameshift mutation in Cloud are shown in bold font.

RT-PCR using primers for Glyma.03G258700 generated fragments of approximately 1 kb length in Harosoy-*T*, Clark-*td,* Cloud, and Williams 82. The coding region of Harosoy-*T* and William 82 was 840 bp long and encoded 279 amino acids (**Figure 3A**). BLAST analysis confirmed that Glyma.03G258700 belongs to a MYB transcription factor of the R2R3 type (**Figure 3A**). A single base was substituted from G to A at the nucleotide position 633 in Clark-*td* compared with Harosoy-*T*. The SNP generated a premature stop codon (TGA) resulting in a short polypeptide consisting of 210 amino acids (**Figure 3B**). Cloud had one base deletion at the nucleotide position 677. This deletion changed a subsequent reading frame and generated a truncated polypeptide consisting of 232 amino acids (**Figure 3B**). Sequencing of the second exon revealed that Korean, Seneca, Kingwa, Grant, and Sooty had the same SNP as Clark-*td*.

**Figure 4:**
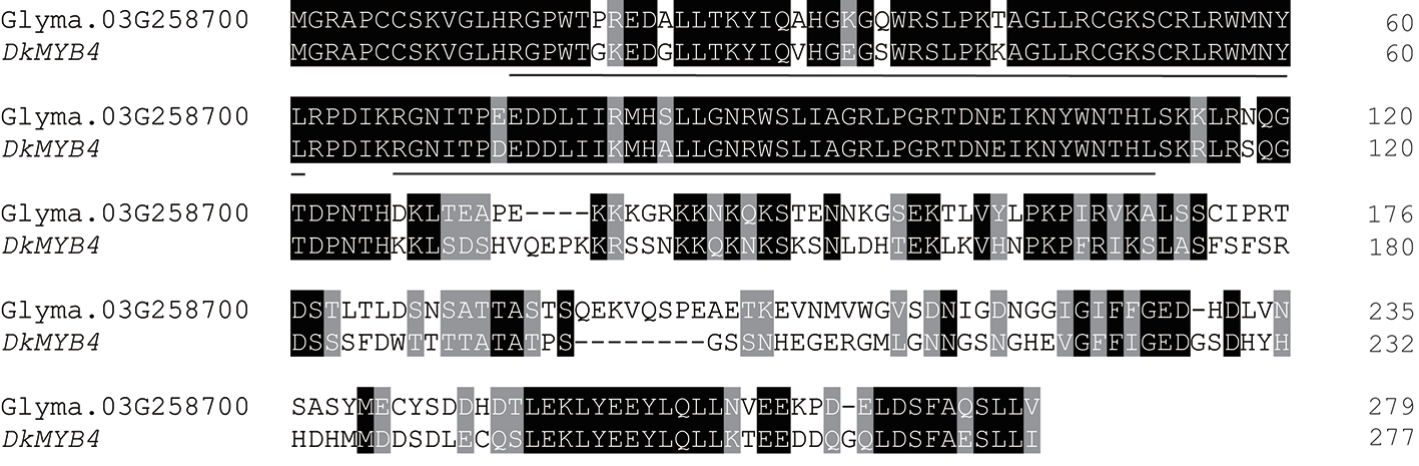
Amino acid alignment of soybean MYB gene, Glyma.03G258700 with persimmon *MYB4* gene, *DkMYB4*. Dashes represent gaps introduced to improve the alignment. Identical and similar amino acids are shown in white font highlighted in black and gray, respectively. MYB DNA-binding domains are underlined.

**Figure 5:**
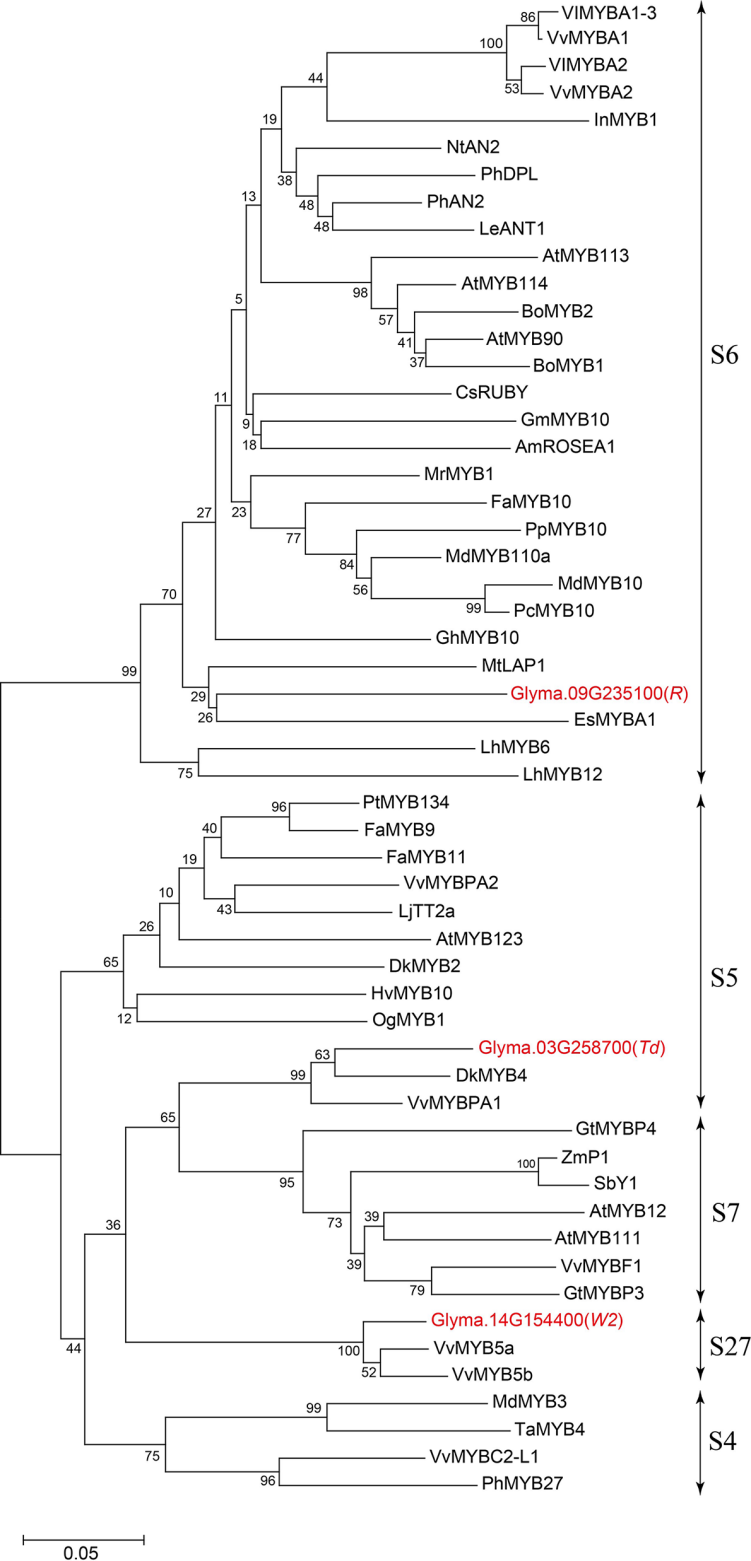
Phylogenetic tree of plant R2R3 MYB transcription factors related to flavonoid biosynthesis. Soybean MYBs are shown in red font. Bar represents 0.05 amino acid substitution/site.

Glyma.03G258700 of Harosoy-*T* had an amino acid identity of 58% with a persimmon MYB gene, *DkMYB4,* which activates proanthocyanidin biosynthesis (Akagi et al., 2009) (**Figure 4**). Particularly, the N-terminal region, including R2R3 MYB-binding domains as well as the C-terminal region, were quite similar. Amino acid identify of the MYB-binding domain was 89%. Plant MYB proteins are classified into 28 subclasses (S1 to S28), and many of them are involved in flavonoid biosynthesis (Liu et al., 2015). In soybean, two flavonoid-related genes, flower color gene *W2* (Glyma.14G154400) and seed coat color gene *R* (Glyma.09G235100), are known to encode R2R3 MYB transcription factors (Gillman et al., 2011; Takahashi et al., 2011; Takahashi et al., 2013; Yan et al., 2015). Based on phylogenetic analysis, Glyma.03G258700 belongs to S5 together with *DkMYB4*, whereas *R* and *W2* genes belong to S6 and S27, respectively (**Figure 5** and **Additional File 2: Table S1**).

### dCAPS Analysis

**Figure 6:**
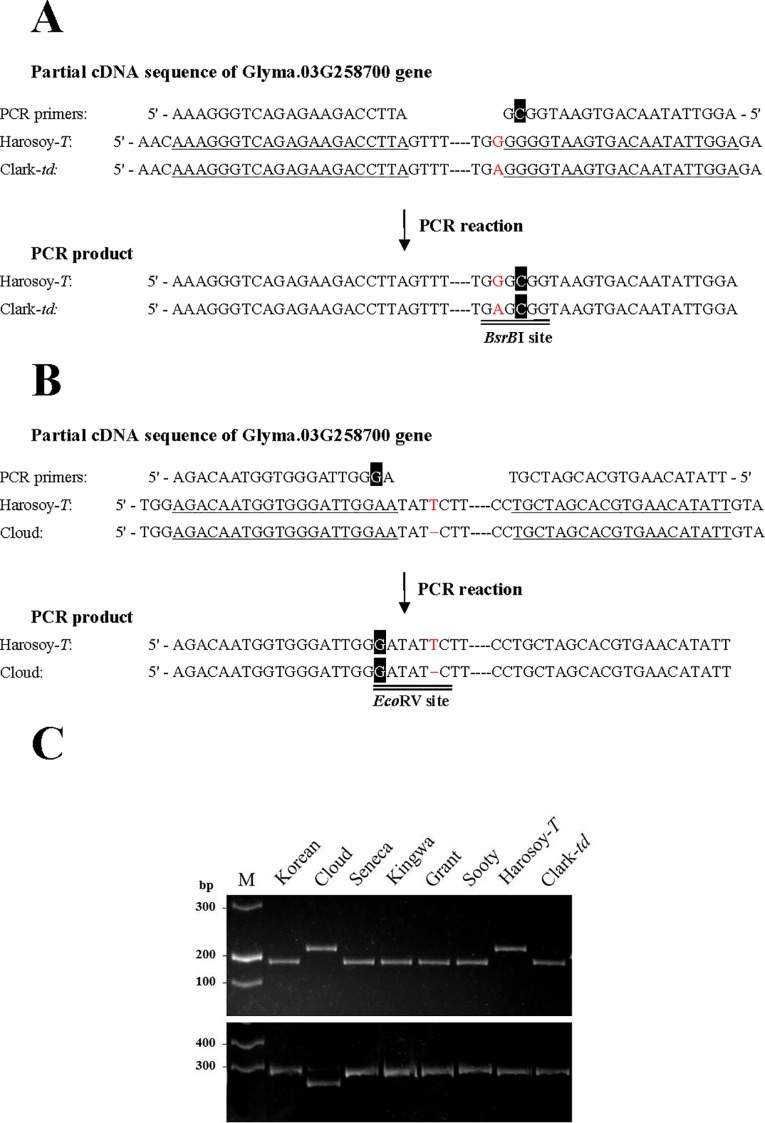
Schematic presentation and results of derived cleaved amplified polymorphic sequences (dCAPS) analysis. **(A)** dCAPS analysis to detect an SNP. A mismatched nucleotide (C, in white font highlighted in black) was incorporated in the reverse primer to generate a *Bsr*BI site in the PCR product of Clark-*td*. **(B)** dCAPS analysis to detect an indel. A mismatched nucleotide (G, in white font highlighted in black) was incorporated in the forward primer to generate an *Eco*RV site in the PCR product of Cloud. Annealing sites of PCR primers are single-underlined. Polymorphic nucleotides are shown in red font. **(C)** Results of dCAPS analysis in pubescence color variants. (Upper panel) Results of dCAPS analysis for SNP. PCR products were digested by *Bsr*BI. (Lower panel) Results of dCAPS analysis for indel. PCR products were digested by *Eco*RV. M: DNA ladder molecular weight marker.

The scheme of derived cleaved amplified polymorphic sequences (dCAPS) analysis for SNP and indel are shown in **Figures 6A**, **B**, respectively. Results of dCAPS analyses are shown in **Figure 6C**. PCR using dCAPS primers for SNP generated amplified products of approximately 210 bp in all genotypes (Korean, Cloud, Seneca, Kingwa, Grant, Sooty, Harosoy-*T*, and Clark-*td*). Digestion with *Bsr*BI generated a fragment of approximately 185 bp in Korean, Seneca, Kingwa, Grant, Sooty, and Clark-*td*, whereas PCR products of Cloud and Harosoy-*T* were undigested. PCR using dCAPS primers for indel generated fragments of approximately 270 bp in all genotypes. Digestion with *Eco*RV generated a fragment of approximately 250 bp in Cloud, whereas PCR products of the other genotypes were unaffected. Thus, Korean, Seneca, Kingwa, Grant, Sooty, and Clark-*td* have the same SNP as PI 157421, whereas Cloud has the same deletion as PI 549046 (**Additional File 3: Figure S2**).

### Gene Expression

**Figure 7:**
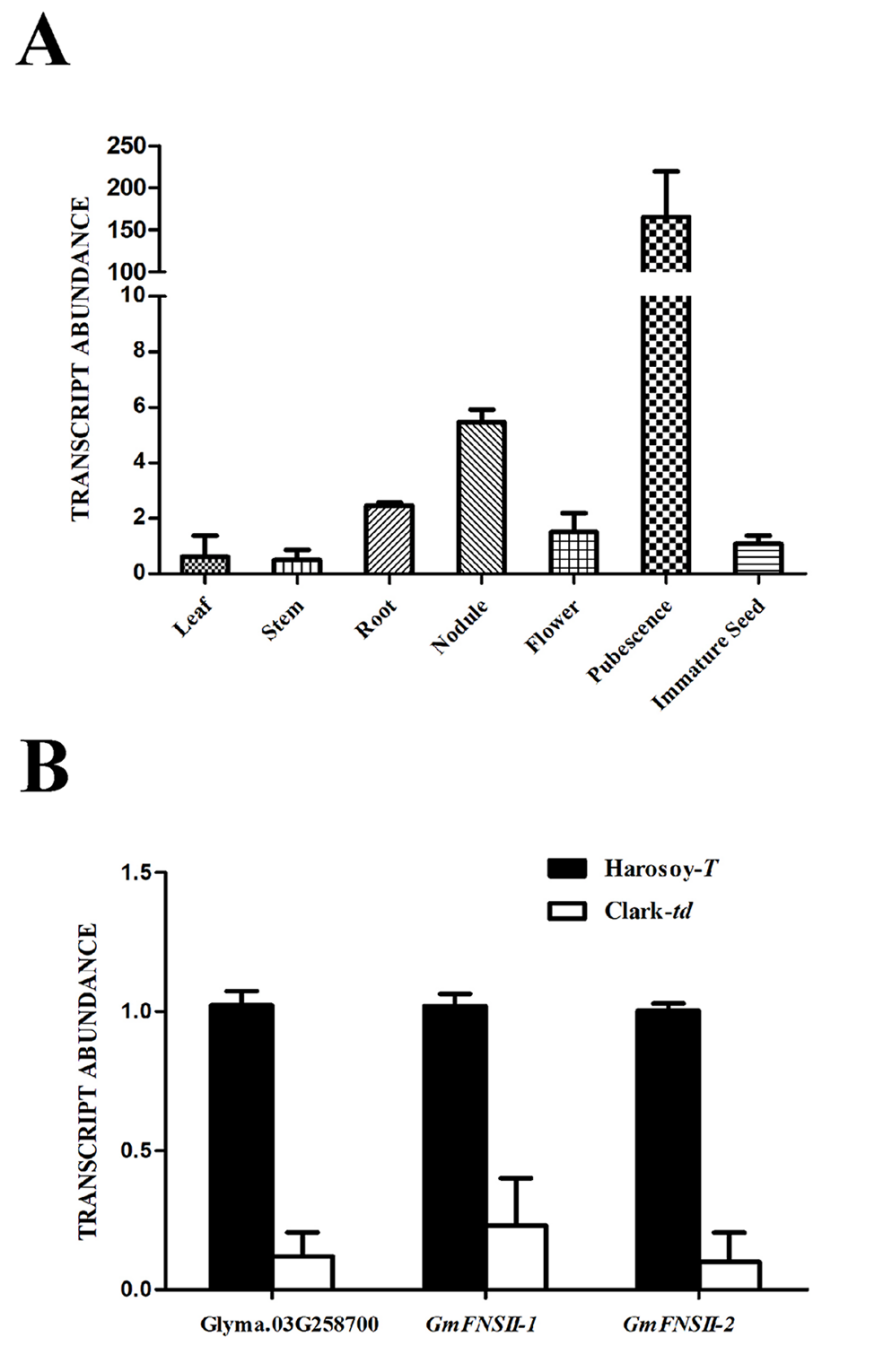
Relative expression of MYB gene (Glyma.03G258700) and two flavone synthase II genes (*GmFNSII-1* and *GmFNSII-2*) in soybean near-isogenic lines. **(A)** Relative expression of Glyma.03G258700 in various tissues of Harosoy-*T*. **(B)** Relative expression of Glyma.03G258700, *GmFNSII-1* and *GmFNSII-2* in pubescence of Harosoy-*T* and Clark-*td*. Transcript levels were standardized by the expression of *actin 1* gene. Means and SDs of three biological replications are shown.

The transcript level of Glyma.03G258700 in pubescence was extremely high (151.6 times of the immature seed), whereas that in the other tissues was comparable with immature seed (leaf: 0.57 time; stem: 0.46 time; root: 2.26 times; root nodule: 5.02 times; flower: 1.38 times) in Harosoy-*T* (**Figure 7A**). In pubescence, the transcript level of Glyma.03G258700 was approximately 12% in Clark-*td* compared with that in Harosoy-*T* (**Figure 7B**). Similarly, the transcript levels of two flavone synthase genes, *GmFNSII-1* and *GmFNSII-2,* in Clark-*td* were approximately 23% and 10%, respectively, of that in Harosoy-*T*.

### Promoter of FNSII Genes

**Figure 8:**
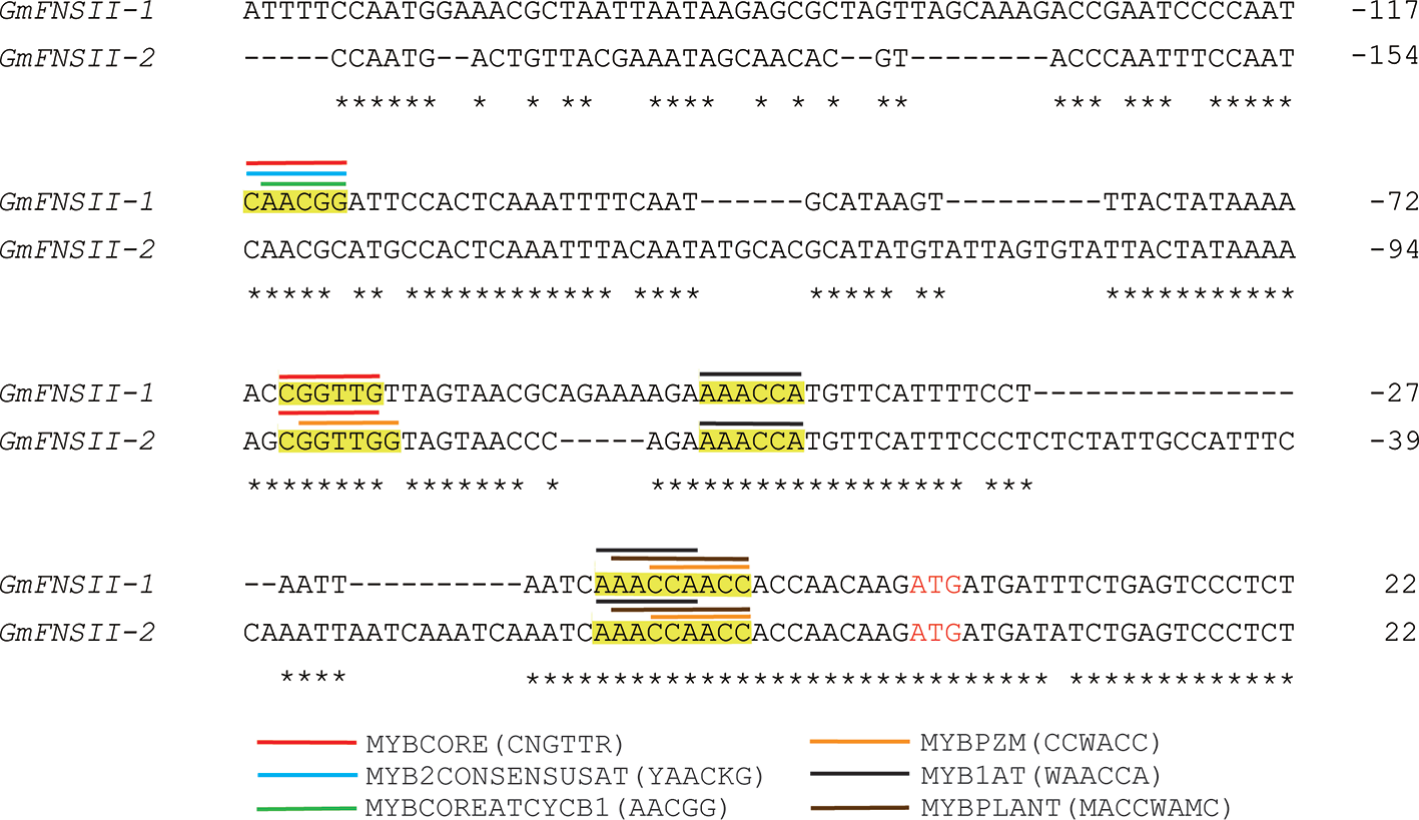
Nucleotide alignment of upstream region of two soybean flavone synthase II genes, *GmFNSII-1* and *GmFNSII-2*, derived from reference genome of cultivar Williams 82. Dashes represent gaps introduced to improve the alignment. Identical nucleotides are indicated by asterisks. Start codon is shown by red font. Putative cis-acting regulatory element regions for MYB transcription factors are highlighted in yellow. Position of each element is shown by bars above nucleotide sequences.

The nucleotide sequence of the promoter region had generally a low identity (18%) among the two flavone synthase II genes, *GmFNSII-1* and *GmFNSII-2,* in Williams 82. However, nucleotides around the end of the region were relatively similar (**Figure 8**). There are six kinds of *cis*-acting regulatory elements for binding of MYB transcription factors (MYBCORE, MYB2CONSENSUSAT, MYBCOREATCYCB1, MYBPZM, MYB1AT, and MYBPLANT) slightly upstream of the coding region, majority of which are shared by the two genes (**Figure 8**).

## Discussion

Pubescence color of soybean is controlled by two genes *T* and *Td* (Bernard, 1975; Palmer et al., 2004). The *T* gene encodes a flavonoid 3ʹ-hydroxylase and hydroxylates the 3ʹ-position of the B-ring to generate dihydroxylated flavonoids (Buttery and Buzzell, 1973; Toda et al., 2002). The dominant *T* allele generates luteolin derivatives and tawny color in pubescence, whereas the recessive *t* allele produces apigenin derivatives and gray color (Iwashina et al., 2006). Thus, *T* gene alters pubescence color by changing the structure of flavones. Under the dominant *T* allele, dominant *Td* allele generates higher amount of luteolin derivatives and tawny color in pubescence (Iwashina et al., 2006). In contrast, recessive *td* allele produces less amount of luteolin derivatives and light tawny (or near-gray) pubescence color. These results suggest that the *Td* gene may control amounts of flavones.

Genetic analysis and linkage mapping using the F_2_ population and F_3_ families derived from a cross between NILs with tawny and light tawny pubescence suggested that a gene responsible for pubescence color was located around the end of chromosome 3, which is consistent with the results of GWAS analysis (Wen et al., 2015; Zhou et al., 2015). Flavones are dependent for their biosynthesis on two functional copies of *FNS II* genes, *FNSII-1* and *FNSII-2*, on chromosome 12 (Fliegmann et al., 2010; Jiang et al., 2010). Accordingly, the *FNS II* genes may not correspond to the *Td* gene. There remains a possibility that the *Td* gene encodes a transcription factor that controls the expression of *FNS II* genes. Genome sequence alignment of plant introductions revealed that near-gray pubescence is associated with premature stop codons in the coding region of Glyma.03G258700 encoding an R2R3 MYB transcription factor.

However, cDNA of Glyma.03G258700 is truncated and lacks a start codon in the reference genome of Williams 82. Genome sequence alignment indicated that no NGS reads were allocated to the 58-nucleotide region in the middle of the first exon. We presume that the reference sequence of Williams 82 has a mistake at this region. We designed a forward primer upstream of the region and successfully cloned the entire cDNA of Harosoy-*T*, Clark-*td*, Cloud, and Williams 82 as well as the entire genomic fragment of Williams 82. The 58-nucleotide sequence of these cDNAs including Williams 82 was completely different from the reference sequence of Williams 82 (**Additional File 1: Figure S1** and **Additional File 3: Figure S2**). Reference sequence of Williams 82 needs to be corrected.

Glyma.03G258700 of Harosoy-*T* consisted of 840 nucleotides and encoded 279 amino acids. In contrast, cDNA of Clark-*td* had a nonsense mutation at the nucleotide position 633 and generated a truncated polypeptide comprising 210 amino acids. Further, Cloud had one base deletion at the nucleotide position 677, resulting in a truncated polypeptide comprising 232 amino acids. dCAPS analysis revealed that Grant, Korean, Kingwa, Sooty, and Seneca with *td* allele had the same SNP with Clark-*td*. Thus, dCAPS analysis confirmed that Seneca with gray pubescence has the *td* allele as expected by pedigree information (Bernard, 1975). R2R3 MYB transcription factors have a conserved DNA-binding domain at the N terminus and a more variable C-terminal transcriptional activation domain (Pireyre and Burow, 2015). These results suggest that Glyma.03G258700 corresponds to the *Td* gene and that premature stop codons disable the transactivating ability of the MYB protein.

There are three types of mutations (type 1 to 3) in the gene (**Figure 2** and **Additional File 3: Figure S2**). Under genetic background of dominant *TT* allele, cultivars of type 2 and 3 have near-gray pubescence whereas type 1 cultivars have either near-gray or light tawny pubescence. Thus, these types were not directly associated with pubescence color, suggesting that other genetic backgrounds may affect the lightness of pubescence color. The expression level of the gene in pubescence of Clark-*td* was very low compared with Harosoy-*T*, probably because of nonsense-mediated mRNA decay, an mRNA surveillance mechanism that eliminates aberrant mRNAs (Chang et al., 2007). Similar observations were made with pubescence color gene *T* (flavonoid 3'-hydroxylase) and flower color gene *Wm* (flavonol synthase) in soybean (Toda et al., 2002; Takahashi et al., 2007).

A large number of R2R3 MYB transcription factors are involved in flavonoid biosynthesis. An additional data file shows that majority of the MYB proteins are involved in biosynthesis of anthocyanins, proanthocyanidins, or flavonols (**Additional File 2: Table S1**) (Liu et al., 2015). The *Td* gene specifically controls color of pubescence, but not changes the color of flower, seed, or hypocotyl (Bernard, 1975; Palmer and Payne, 1979). The allele at the *Td* locus did not affect the amounts of anthocyanins, flavonol glycosides, or dihydroflavonol in petals (Iwashina et al., 2007). In addition, the present study revealed that Glyma.03G258700 expresses predominantly in pubescence where flavones are almost exclusively deposited. These results suggest a hypothesis that Glyma.03G258700 has evolved to upregulate biosynthesis of flavones existing in pubescence. Further investigation including expression assays of other flavonoid biosynthesis genes may be necessary to ascertain this hypothesis.

Expression levels of Glyma.03G258700, *GmFNSII-1,* and *GmFNSII-2* in pubescence of Clark-*td* were extremely low compared with those in Harosoy*-T.* In addition, the promoter region of *FNSII-1* and *FNSII-2* shared *cis*-regulatory elements for MYB transcription factors, including the MYBCORE element. Persimmon DkMYB4 protein having a quite similar MYB-binding domain was proven to bind to the MYBCORE element by electrophoretic mobility shift assays (Akagi et al., 2009). These results strongly suggest that the Glyma.03G258700 protein may also bind to a similar element in the promoter of *FNSII* genes and upregulate their transcription.

In summary, the wild-type Glyma.03G258700 protein may bind to the promoter of *FNS II* genes and upregulate their transcription, resulting in high flavone amounts and tawny pubescence color. In contrast, premature stop codons in Glyma.03G258700 may cause a dysfunction in the transactivating ability of the MYB protein. The mutated proteins fail to promote the expression of *FNSII* genes, resulting in lower flavone amounts and dilute pubescence color. *FNS II* converts flavanones to flavones. Clark-*td* occasionally produces isoflavonoids in pubescence (Iwashina et al., 2006). Mutated MYB protein may have failed to promote catalysis of flavanones to flavones. The remaining flavanones may have been transformed by isoflavone synthase to generate isoflavonoids. Electrophoretic mobility shift assays may be necessary to ascertain the interaction between the Glyma.03G258700 protein and the *cis*-acting regulatory elements in the promoter of *FNSII* genes. Gene editing and transgenic experiments to prove our hypothesis that Glyma.03G258700 corresponds to the *Td* gene, upregulates flavone biosynthesis, and deepens pubescence color, remain as future work. Investigations of its promoter may help identify *cis*-acting regulatory elements involved in pubescence-specific gene expression and develop a genetic system to overproduce specific proteins in the pubescence of soybean.

## Conclusion

This study revealed that soybean *Td* gene encodes a unique R2R3 MYB transcription factor, which controls flavone content and pubescence color. The wild type of MYB protein binds to the promoter of *FNS II* genes and upregulates their expression, resulting in higher flavone content and deeper pubescence color. Loss-of-function mutation of the MYB gene fails to promote expression of *FNSII* genes, resulting in diluted pubescence color.

## Data Availability Statement

Sequence data from this article have been deposited to DDBJ, with the accession numbers LC485152, LC485153, LC485154, LC512739 and LC512740. The datasets used and/or analyzed during the current study are available from the corresponding author on reasonable request.

## Author Contributions

FY and RT designed experiments. SG did genetic analysis and linkage mapping. FY, YL, YS, and QW carried out molecular cloning, dCAPS analysis, and gene expression assays.

## Funding

FY is supported by National Key Research Project (2017YFD0101304), National Natural Science Foundation of China (31671714), Science and Technology Department of Jilin Province (20180201030), and the Fundamental Research Funds for the Central Universities. SG is funded by the Invitational Fellowships for Research in Japan (L08553) (Japan Society for the Promotion of Science).

## Conflict of Interest

The authors declare that the research was conducted in the absence of any commercial or financial relationships that could be construed as a potential conflict of interest.

## Supplementary Material

Additional File 1: Figure S1 Comparison of nucleotide sequence around the 1st exon of soybean Glyma.03G258700 among reference genome of cultivar Williams 82 (Wm82.a2.v1), genome of three plant introductions and cultivar Bay, and cDNA of Harosoy-*T*. Genome alignment was created by the Integrative Genomics Viewer. Nucleotide position in chromosome 3 is indicated by arrows above panel. Genome sequence of Williams 82 is shown below panel and aligned with cDNA of Harosoy-*T*. Identical nucleotides are shown by asterisks. PCR primer for cDNA cloning is single-underlined. Start codon is double-underlined. Exon and intron of Harosoy-*T* are drawn by black box harboring amino acids and black line, respectively. Gene orientation is indicated by an arrow underneath. Only amino acids in yellow font are indicated as 1st exon in Wm82.a2.v1.

Additional File 2: Table S1 List of R2R3 MYB transcription factors used in phylogenetic analysis.

Additional File 3: Figure S2 Gene structure of Glyma.03G258700 deduced from sequencing or dCAPS analysis in pubescence color variants. Exons and introns are shown by boxes and lines, respectively. White and black triangles indicate start codons and stop codons, respectively. Red box represents a region where frameshift mutation occurred due to deletion. Numbers indicate nucleotide position starting from second exon.
